# Patterns and Trajectories of Pulmonary Function in Coronavirus Disease 2019 Survivors: An Exploratory Study Conducted in Central India

**DOI:** 10.7759/cureus.26955

**Published:** 2022-07-17

**Authors:** Rachna Parashar, Ankur Joshi, Pragati Raghuwanshi, Rajnish Joshi, Sandip Hulke, Jai Prakash Sharma

**Affiliations:** 1 Physiology, All India Institute of Medical Sciences, Bhopal, Bhopal, IND; 2 Community and Family Medicine, All India Institute of Medical Sciences, Bhopal, Bhopal, IND; 3 General Medicine, All India Institute of Medical Sciences, Bhopal, Bhopal, IND; 4 Anesthesiology, All India Institute of Medical Sciences, Bhopal, Bhopal, IND

**Keywords:** covid-19, coronavirus, comorbidity, spirometry, body mass index

## Abstract

Background: The ongoing pandemic of coronavirus disease 2019 (COVID-19) has negatively impacted respiratory health worldwide. The severity of the disease varies considerably, and patients may present with bronchitis, pneumonia, and acute respiratory distress syndrome. This study aims to quantify the parameters of the pulmonary function test (PFT) with regard to the severity of COVID-19 and understand the pattern of PFT in reference to the status of selected morbidities and body mass index.

Materials and methods: This is a hospital-based, comparative, cross-sectional study. A total of 255 COVID-19 survivors underwent clinical assessment, a PFT, and a 6-minute walk test. Participants were divided into mild, moderate, and severe disease groups. The parameters were compared between these groups. The PFT and 6-minute walk tests were conducted using an NDD Digital computerized spirometer (NDD Meditechnik AG., Switzerland) and a fingertip pulse oximeter (Hasely Inc., India), respectively.

Results: All PFT parameters showed significant differential distribution among the severity groups (p<0.001) except for forced expiratory volume in 1 s/ forced vital capacity (FEV_1_/FVC) and forced expiratory flow (FEF) during 25%-75% expiration and peak expiratory flow (PEF). Among severe category participants forced vital capacity (FVC), forced expiratory volume in one second (FEV_1_), and FEV_1_/FVC, were significantly reduced as compared to mild and moderate. Severity was significantly affected by age >50 years. Severe category participants were seen in 31% of normal, 58% of pre-obese, and 53% of obese participants; however, this difference was insignificant. A significant reduction in SPO2 on the 6-minute walk test was observed in severely sick participants.

Conclusions: COVID-19 is associated with a mixed pattern of spirometry. Poor prognosis is associated with older age, obesity, and multimorbidity.

## Introduction

The coronavirus disease 2019 (COVID-19) pandemic, caused by severe acute respiratory syndrome coronavirus 2 (SARS-CoV-2), has affected >367 million individuals and resulted in >5 million deaths worldwide [1]. The disease primarily affects the lungs and other respiratory organs. Damage to the lungs results from a cytokine storm induced by the host's immune system. This immune response leads to acute inflammation and increased levels of inflammatory biomarkers, such as C-reactive protein, ferritin, and interleukin-6 (IL-6) [2]. Based on the widely documented lung injuries related to COVID-19, concerns are raised regarding assessing lung injury in discharged patients [3]. The severity of clinical manifestations ranges considerably, and patients present with bronchitis, pneumonia, and acute respiratory distress syndrome (ARDS). COVID-19-associated ARDS has been linked to poor prognosis [4]. A follow-up study of 81 patients with COVID-19 through computed tomography revealed a reticular pattern associated with bronchiectasis and irregular interlobular or septal thickening. These findings indicate the appearance of interstitial changes, suggesting the development of fibrosis [5].

Another follow-up study by Fumagalli et al. revealed deranged pulmonary functions in a restrictive pattern [6]. Nevertheless, Thomas et al. suggested that all spirometry parameters were normal except the changes were observed in diffusing capacity with a worse impact on those with severe disease [7]. Owing to the complex pathophysiology, the status of the lungs and degree of recovery remains unknown [8]. According to the available evidence on the COVID-19 pandemic, there is limited knowledge regarding the effects of the virus in terms of residual changes or long-term sequelae [9-12]. Notably, the pulmonary function test (PFT) and spirometry are important, reliable, and easy to perform tools for the diagnosis and management of respiratory disease in all age groups. Hence, they are used in the present study to screen residual pulmonary changes in COVID-19 survivors. Moreover, disease progression is a complex phenomenon and bi-directionally affected by protective (e.g., immune status, good nutrition) or deleterious (e.g., obesity, comorbidity) effects.

According to guidelines established by the World Health Organization, the presentation and progression of illness are categorized into the following groups: mild; moderate; and severe [13]. Moreover, it has been found that disease progression and severity are associated with the presence of comorbidities [14]. As obesity is one of the comorbidity conditions its high prevalence rate may directly or indirectly interfere with the treatment and prognosis of ARDS. This condition poses challenges due to its unique physiology in patients and, indirectly, is a risk factor for the development of chronic obstructive pulmonary disease (COPD) [15]. Hence, investigating the long-term sequelae of COVID-19 in survivors is urgently warranted [16]. Currently, evidence regarding the pattern of COVID-19 residual changes among survivors during the recovery phase, as well as its correlation with the comorbidity status and body mass index (BMI), is scarce [17-19].

Thus, the aim of this study was to analyze the patterns and trajectories of COVID-19 in pulmonary functions of survivors classified into different clinical categories.

## Materials and methods

Participants and study design

This was a hospital-based, comparative, cross-sectional study. Ethical approval was obtained from the institutional ethical committee (approval number: IHEC-LOP/2020/EF0219). A total of 546 COVID-19 survivors were followed up at the Outpatient Department of the All India Institute of Medical Sciences (Bhopal, India). Of those, 255 provided written informed consent and met the inclusion criteria: age 18-65 years and discharge from hospital ≥2 weeks prior to inclusion in the study. Participants were enrolled between July 2021 and September 2021.

Data collection

All the precautions related to the COVID-19 care protocol were implemented during this study. Clinicians performed PFTs after recording a detailed history of the patient and conducting a clinical assessment. Patient characteristics (e.g., body mass index {BMI}, comorbidity status, smoking habits, signs and symptoms, and other demographic information) were collected. Based on clinical and treatment history, and according to the World Health Organization performance scale, the enrolled COVID-19 survivors were divided into the following disease severity groups: mild; moderate; and severe. The enrolled survivors were categorized in accordance with the history of hospitalization, requirement of supplemental oxygen, and requirement for admission to the intensive care unit [13].

Clinical assessment included the 6-minute walk test (6MWT) using a fingertip pulse oximeter (Hasely Inc., India). This analysis was performed at room temperature under the supervision of a respiratory therapist. Oxygen saturation (SpO2) absolute values were categorized from 0 to 2, as per the relative capability to perform the 6MWT.

The BMI (weight/height {kg/m^2^}) of all enrolled survivors was also determined, and the patients were categorized as normal, obese, and pre-obese (18-21, <25, and 22-25 kg/m^2^, respectively) [20]. In addition, the presence of comorbidities (i.e., diabetes, hypertension, COPD, and multimorbidity) was evaluated.

Measurement of PFT

PFT was performed in a sitting position using an NDD Digital computerized spirometer (NDD Meditechnik AG., Switzerland). Prior to undergoing the test, the participants were familiarized with the instrument and the procedure. PFT parameters were considered according to the maneuver-acceptable criteria established by the American Thoracic Society and the European Respiratory Society [21]. PFT parameters were recorded as best trial and percentage. PFT parameters included the following: forced vital capacity (FVC); forced expiratory volume in 1 s (FEV_1_); FEV_1_ as a percentage of the FVC; peak expiratory flow rate; forced expiratory flow rate during 25-75% of expiration (FEF25-75); and forced inspiratory vital capacity (FIVC).

Statistical analysis

Data were validated for redundancies and missing values, and they were descriptively summarized using the mean, median, and interquartile ranges (in the case of non-Gaussian distribution). Categorized PFT values were compared through an unpaired analysis of variance test to analyze the shift of categorized PFT distribution values from null parent distributions. Proportional stack diagrams were created to illustrate the effect of BMI categories on PFT values and the direction of possible interactions.

## Results

Participant characteristics

A total of 255 COVID-19 survivors were enrolled in this study (167 males and 88 females; mean age: 47.12 ± 13.78 years). Of those, participants were classified into the mild, moderate, and severe disease groups as shown in Table 1. The distribution of the baseline characteristics of enrolled COVID-19 survivors was categorized according to disease severity presented as the mean (±standard deviation) or numbers (%). The comorbidities mainly presented with diabetes and hypertension were found to be statistically significantly distributed (p = <0.001), except for COPD (p=0.7). A significant distribution was also found with reference to their multimorbidity status. 

**Table 1:** Baseline characteristics of enrolled COVID-19 survivors categorized according to disease severity ^1^Kruskal–Wallis rank-sum test; Pearson's chi-squared test; Fisher's exact test. Data are presented as the mean (±standard deviation) or numbers (%). *p < 0.05, statistically significant.

Characteristic	Overall N = 255	Mild N = 42	Moderate N = 82	Severe N = 131	p-value^1^
Age, years	47.12 (±13.78)	41.60 (±14.12)	42.88 (±13.54)	51.54 (±12.38)	<0.001*
Sex					0.2
Female	88/88 (100%)	14/88 (16%)	22/88 (25%)	52/88 (59%)	
Male	167/167 (100%)	28/167 (17%)	60/167 (36%)	79/167 (47%)	
Diabetes					<0.001*
Absent	155/155 (100%)	39/155 (25%)	67/155 (43%)	49/155 (32%)	
Present	100/100 (100%)	3/100 (3.0%)	15/100 (15%)	82/100 (82%)	
Hypertension					0.002*
Absent	162/162 (100%)	33/162 (20%)	59/162 (36%)	70/162 (43%)	
Present	93/93 (100%)	9/93 (9.7%)	23/93 (25%)	61/93 (66%)	
Chronic obstructive pulmonary disease					0.7
Absent	240/240 (100%)	41/240 (17%)	76/240 (32%)	123/240 (51%)	
Present	15/15 (100%)	1/15 (6.7%)	6/15 (40%)	8/15 (53%)	
Multimorbidity					<0.001*
Absent	194/194 (100%)	40/194 (21%)	69/194 (36%)	85/194 (44%)	
Present	61/61 (100%)	2/61 (3.3%)	13/61 (21%)	46/61 (75%)	
Body mass index	26.40 (±4.37)	25.61 (±4.08)	26.08 (±4.44)	26.84 (±4.40)	0.4
Place of care					<0.001*
Home isolation	42/42 (100%)	41/42 (98%)	1/42 (2.4%)	0/42 (0%)	
Hospital/ward	157/157 (100%)	1/157 (0.6%)	81/157 (52%)	75/157 (48%)	
Hospital/intensive care unit	56/56 (100%)	0/56 (0%)	0/56 (0%)	56/56 (100%)	

PFT characteristics

All parameters exhibited a differential distribution among the disease severity groups. These differences were statistically significant, except for the FEV_1_/FVC ratio (p = 0.079). The mean values of FVC, FEV_1_, FEF25-75, PEF, FET, FIVC, and PIF parameters were lower in the disease severity group as compared to the mild and moderate groups, which suggests the restrictive pattern (Table 2).

**Table 2:** Best trail PFT parameters of enrolled COVID-19 survivors ^1^Kruskal–Wallis rank-sum test. COVID-19, coronavirus disease-2019; FEF25–75, forced expiratory flow during 25–75% of expiration; FET, forced expiratory time; FEV_1_, forced expiratory volume in 1 s; FIVC, forced inspiratory vital capacity; FVC, forced vital capacity; PEF, peak expiratory flow; PFT, pulmonary function test; PIF, peak inspiratory flow rate. Data are presented as the mean (±standard deviation) or numbers (%). *p < 0.05, statistically significant.

Characteristic (best trial)	Overall N = 255	Mild N = 42	Moderate N = 82	Severe N = 131	p-value^1^
FVC	2.86 (±5.05)	3.02 (±0.91)	3.06 (±0.80)	2.68 (±7.01)	<0.001*
FEV_1_	2.13 (±0.79)	2.50 (±0.81)	2.56 (±0.71)	1.75 (±0.62)	<0.001*
FEV_1_/FVC	83.15 (±11.93)	80.03 (±15.73)	83.63 (±7.23)	83.85 (±12.80)	0.079
FEF_25–75_	2.69 (±1.22)	2.83 (±1.26)	3.08 (±1.27)	2.40 (±1.10)	<0.001*
PEF	5.66 (±2.39)	6.02 (±2.23)	6.25 (±2.15)	5.17 (±2.48)	<0.001*
FET	4.64 (±2.08)	5.04 (±2.19)	5.10 (±2.07)	4.22 (±1.98)	0.003*
FIVC	2.20 (±1.22)	2.65 (±1.38)	2.75 (±1.13)	1.71 (±1.01)	<0.001*
PIF	3.22 (±1.90)	3.63 (±1.92)	3.62 (±1.85)	2.83 (±1.86)	<0.001*

All parameters showed a differential distribution among the disease severity groups. These differences were statistically significant, except for FEF25-75 (p = 0.2) (Table 3).

**Table 3:** PFT parameter percentage (%) of their predicted values of enrolled COVID-19 survivors ^1^Kruskal–Wallis rank-sum test. COVID-19, coronavirus disease-2019; FEF25–75, forced expiratory flow during 25%–75% of expiration; FEV_1_, forced expiratory volume in 1 s; FIVC, forced inspiratory vital capacity; FVC, forced vital capacity; PEF, peak expiratory flow; PFT, pulmonary function test. Data are presented as the mean (±standard deviation) or numbers (%). *p < 0.05, statistically significant.

Characteristic (%)	Overall N=255	Mild N=42	Moderate N=82	Severe N=131	p-value^1^
FVC	68.68 (±18.06)	77.79 (±16.09)	76.52 (±14.49)	60.85 (±17.26)	<0.001*
FEV_1_	69.80 (±18.25)	77.93 (±18.38)	77.10 (±15.33)	62.63 (±17.05)	<0.001*
FEV_1_/FVC	106.37 (±10.80)	103.05 (±11.29)	104.52 (±9.21)	108.59 (±11.14)	0.002*
FEF_25–75_	71.38 (±27.47)	70.21 (±29.17)	76.05 (±26.45)	68.82 (±27.38)	0.2
PEF	73.37 (±26.28)	74.00 (±26.81)	77.70 (±24.82)	70.47 (±26.79)	0.093
FIVC	59.60 (±28.19)	66.34 (±32.92)	69.34 (±23.94)	51.33 (±26.70)	<0.001*

We further classified the enrolled COVID-19 survivors into binary groups by setting the cut-off value at 80% for FVC, FEV_1_, and the FEV_1_/FVC ratio. This classification represents the distribution of obstructive and restrictive changes according to the severity of the disease (Tables 4-6).

**Table 4:** Categorical comparison of FVC with disease severity ^1^Pearson's chi-squared test. FVC, forced vital capacity. Data are presented as numbers (%). *p < 0.05, statistically significant.

Characteristic	FVC <80%	FVC >80%	Total	p-value^1^
Severity groups				<0.001*
Mild	25 (60%)	17 (40%)	42 (100%)	
Moderate	49 (60%)	33 (40%)	82 (100%)	
Severe	119 (91%)	12 (9.2%)	131 (100%)	
Total	193 (76%)	62 (24%)	255 (100%)	

**Table 5:** Categorical comparison of FEV1 with disease severity ^1^Pearson's chi-squared test. FEV_1_, forced expiratory volume in 1 s. Data are presented as numbers (%). *p < 0.05, statistically significant.

Characteristic	FEV_1_ <80%	FEV_1_ >80%	Total	p-value^1^
Severity groups				<0.001*
Mild	20 (48%)	22 (52%)	42 (100%)	
Moderate	46 (56%)	36 (44%)	82 (100%)	
Severe	111 (85%)	20 (15%)	131 (100%)	
Total	177 (69%)	78 (31%)	255 (100%)	

**Table 6:** Categorical comparison of the FEV1/FVC ratio with disease severity ^1^Fisher's exact test. FEV_1_/FVC, forced expiratory volume in 1 s/forced vital capacity. Data are presented as numbers (%). *p < 0.05, statistically significant.

Characteristic	FEV_1_/FVC > 80%	FEV_1_/FVC< 80%	Total	p-value^1^
Severity groups				0.043
Mild	39 (93%)	3 (7.1%)	42 (100%)	
Moderate	79 (96%)	3 (3.7%)	82 (100%)	
Severe	130 (99%)	1 (0.8%)	131 (100%)	
Total	248 (97%)	7 (2.7%)	255 (100%)	

We observed an increase in the number of participants in the <80% group based on the disease severity. This may denote an association of severity with the parameters examined in this study.

We further investigated the potential effects of age and BMI on disease severity as per their category (Table 7) that suggested the presence of disease in severe form among higher age group individuals. The mean 6-minute walking distance SpO2 in all participants was above 96% except for the disease severity group who had a low 6MWDs SpO2 mean, 94.99 (±3.29), and it was also statistically significant distributed (p = <0.001).

**Table 7:** Effect of age and BMI on disease severity in COVID-19 survivors ^1^Kruskal–Wallis rank-sum test; Pearson’s chi-squared test; Fisher’s exact test. BMI, body mass index; COVID-19, coronavirus disease-2019; SpO2, oxygen saturation. Data are presented as the mean (±standard deviation) or numbers (%). *p < 0.05, statistically significant.

Characteristic	Overall N = 255	Mild N = 42	Moderate N = 82	Severe N = 131	p-value^1^
6-min SpO_2_	96.27 (±2.98)	97.90 (±1.59)	97.48 (±1.91)	94.99 (±3.29)	<0.001*
Age groups					<0.001*
<30 years	33/33 (100%)	10/33 (30%)	19/33 (58%)	4/33 (12%)	
30–50 years	107/107 (100%)	21/107 (20%)	35/107 (33%)	51/107 (48%)	
>50 years	115/115 (100%)	11115 (9.6%)	28/115 (24%)	76/115 (66%)	
BMI groups					0.085
Normal	35/35 (100%)	8/35 (23%)	16/35 (46%)	11/35 (31%)	
Pre-obese	67/67 (100%)	12/67 (18%)	16/67 (24%)	39/67 (58%)	
Obese	153/153 (100%)	22/153 (14%)	50/153 (33%)	81/153 (53%)	

**Figure 1:**
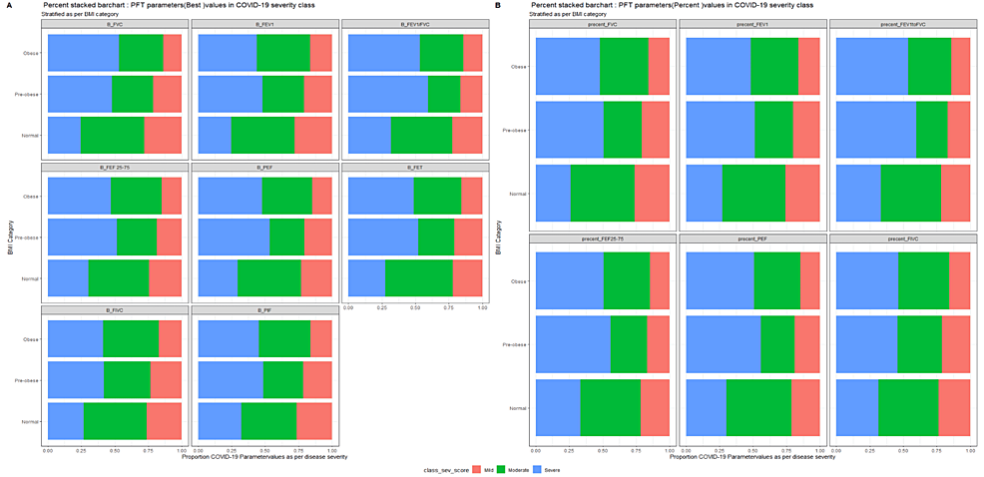
Effect of multimorbidity on COVID-19 severity according to the PFT parameters of survivors. COVID-19, coronavirus disease-2019; FEF25–75, forced expiratory flow during 25%–75% of expiration; FEV_1_, forced expiratory volume in 1 s; FIVC, forced inspiratory vital capacity; FVC, forced vital capacity; PEF, peak expiratory flow; PFT, pulmonary function test. The enrolled COVID-19 survivors were divided into disease severity groups and represented as: mild = red; moderate = green; and severe = blue.

**Figure 2:**
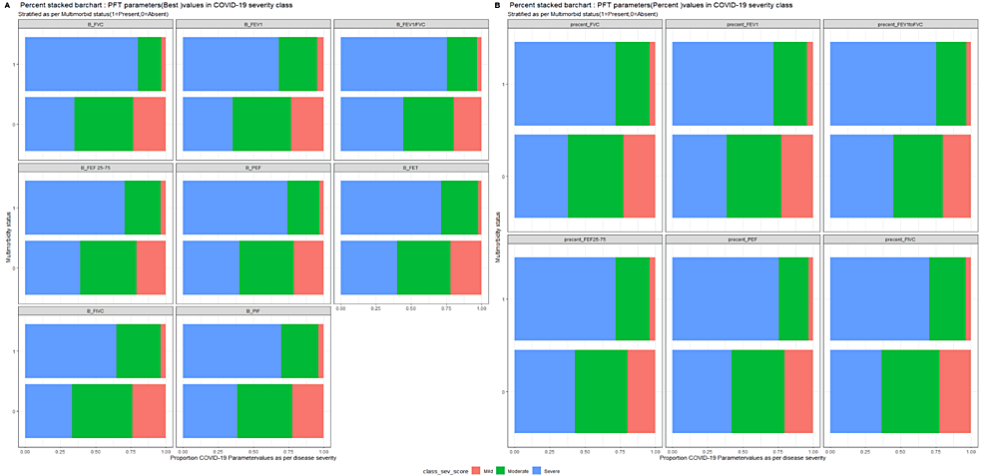
Effect of BMI on COVID-19 severity according to the PFT parameters of survivors. BMI, body mass index; COVID-19, coronavirus disease-2019; FEF25–75, forced expiratory flow during 25%–75% of expiration; FEV_1_, forced expiratory volume in 1 s; FIVC, forced inspiratory vital capacity; FVC, forced vital capacity; PEF, peak expiratory flow; PFT, pulmonary function test. The enrolled COVID-19 survivors were divided into disease severity groups and represented as: mild = red; moderate = green; and severe = blue.

The effect of multimorbidity and BMI according to the PFT parameters of COVID-19 survivors are illustrated with the help of a stake diagram (Figures 1, 2).

## Discussion

Currently, there is a scarcity of large-scale studies focusing on changes in pulmonary function among COVID-19 survivors. Using spirometry, this study focused on patterns of changes in pulmonary functions in relation to morbidity and disease severity.

COVID-19 survivors aged 18-65 years were enrolled in the present study. Those in the older age group (i.e., >50 years) associated with severe disease are 76 (66%) out of 115; moreover, 56 of the 131 severe cases (42%) required intensive care management. Hence, the findings were suggestive of a link between severe disease and advanced age. This association may be due to the higher rates of comorbid conditions present in older patients [22].

Our study also revealed that PFT parameters were significantly associated with disease severity. The detected changes were both obstructive and restrictive, suggesting a mixed pattern of long-term sequelae of COVID-19. Significant changes were not found in FEV_1_/FVC, FEF25-75, and peak expiratory flow. A similar study conducted by Fumagalli et al., involving a smaller sample size, suggested mainly restrictive changes in COVID-19 survivors [6].

In the present study, patterns of changes in PFT parameters were also studied using a binary category setting and a cut-off value of 80% [21]. Impairment of pulmonary function in the obstructive pattern (FEV_1_) was found in 177 (69%) participants out of 255. In the restrictive pattern (FVC), this rate was in 76% (19) of participants. In the obstructive form, 20 (48%) and 111 (85%) of the enrolled COVID-19 survivors presented mild and severe disease, respectively. In the restrictive changes, these rates were observed in 60% (25) and 91% (119) participants, respectively. This evidence indicates that the rate of restrictive changes was slightly higher than that of obstructive changes. The findings of our study are in coherence with the previously reported occurrence of progressive fibrosis as a consequence of ARDS [23].

Using computed tomography, Zhou et al. revealed that diffuse pulmonary dysfunction was common, with a high incidence of pulmonary sequelae regardless of disease severity [3]. This is attributed to diffuse alveolar damage, severe endothelial injury, widespread thrombosis with microangiopathy, alveolar septal fibrous growth, and pulmonary consolidation, as well as lower lung elasticity in critically ill patients.

According to the American Thoracic Society and the European Respiratory Society, the FEV_1_/FVC ratio is the most sensitive PFT parameter. In almost all COVID-19 survivors (97%) this ratio was >80%; 99% of these had severe disease [21]. This suggests that none of the enrolled COVID-19 survivors were in respiratory distress during the PFT. Alternatively, this result could be due to the fact that residual changes were slightly more restrictive than obstructive.

To verify this, we further investigated the results of the 6MWT and SpO2, which were analyzed using a score ranging from 0 to 2. We found a score of 2 and SpO2 >96%, which suggested that study participants were not in respiratory distress at the time of the study [24].

In the analysis of the PFT results, it was observed that 193 of 255 participants were hospitalized with comorbidities. The most frequent comorbidities were diabetes in 100 participants (39.21%), hypertension in 93 participants (36.47%), and multimorbidity in 61 participants (23.98%). We found a significant association of disease severity with diabetes and hypertension, but not COPD. Notably, participants with multiple comorbidities were at higher risk of severe COVID-19 versus those with single comorbidity [22].

Older participants and those with higher BMI were associated with a poor prognosis; thus, they require early and intensive care [25]. Of note, the effects of COPD and BMI were not significantly associated with pulmonary function. Importantly, there was a link between the long-term sequelae of COVID-19 and multimorbidity, particularly diabetes and hypertension.

Strength and limitation

The inferences drawn in this study were based on data derived from the disease severity groups and various PFT parameters. The participants were further classified into several groups to address the effect of possible confounders and effect modifiers. However, considering the limitations of cross-sectional studies, it may be difficult to comment with certainty. One should be cautious while drawing the causal inference for which serial measurements and adjustment through multivariate models might serve the purpose.

## Conclusions

Based on the present findings, we may conclude that COVID-19 is associated with a mixed pattern of spirometry. As measured by spirometry, older adults with diabetes Mellitus and hypertension are associated with deterioration in respiratory functions. We recommend that these patients must take extra precautions against COVID-19. Periodic follow-up for appropriate timely treatment and rehabilitation is advised in such patients. We recommend various multi-centric trials regarding the role of rehabilitation programs in form of yoga/exercise in COVID-19 patients with various comorbidities.
